# Effects of 8-Week High-Intensity Interval Training Intervention Regulating the SIRT1/PGC1α Pathway on Hippocampal Neuron Injury and Cognitive Impairment in Obese Rats

**DOI:** 10.1016/j.cdnut.2025.107548

**Published:** 2025-09-04

**Authors:** Kaiyin Cui, Jiabao Zhang, Huiting Wei, Yifu Meng, Changshuo Li, Ruizhe Liao, Jiajie Miao, Hao Su

**Affiliations:** 1 The Graduate School, Beijing Sport University, Beijing, China; 2 The School of Sports Science, Beijing Sport University, Beijing, China; 3 Key Laboratory of Exercise and Physical Fitness (Beijing Sport University), Ministry of Education, Beijing, China; 4 Beijing Higher School Engineering Research Center of Sport Nutrition, Beijing, China

**Keywords:** HIIT, obesity, CI, SIRT1/PGC1α

## Abstract

**Background:**

Obesity can causes changes in cognitive function, leading to cognitive impairment (CI).

**Objectives:**

This study aimed to explore the effects of high-intensity interval training (HIIT) on hippocampal neuronal damage, cognitive function, and the SIRT1/PGC1α pathway in obese rats and provide a theoretical basis for HIIT intervention in improving CI caused by obesity.

**Methods:**

Rats with successful obesity modeling and rats of the same age were randomly divided into a normal quiet group (CSG, *n* = 10), regular exercise group (CEG, *n* = 10), high-fat quiet group (HSG, *n* = 10), and high-fat exercise group (HEG, *n* = 10). Rats in the exercise group underwent an 8-week (8-wk) HIIT training. Subsequently, behavioral testing and sampling indicator testing were conducted.

**Results:**

Compared with the normal quiet group, the exercise group showed a significant decrease in body weight and Lee index, whereas the obesity group showed a significant increase. The Morris water maze experiment showed that compared with the CSG, the HSG had a longer latency period and a reduced number of platform crossings. The latency period of the CEG was shortened, and the frequency increased. Compared with the HSG, the HEG had a shorter latency period and an increase in frequency. Organizational staining showed that the HSG had reduced neuron number, deepened staining, chaotic arrangement, and Nissl body lysis in the hippocampal CA1 region, whereas HIIT improved these pathological changes. RT-qPCR showed obesity reduces the mRNA level of *Sirt1* gene in hippocampal tissue, whereas exercise increases it. Western blot analysis showed exercise and obesity independently regulate the SIRT1/PGC1α pathway: exercise upregulates its expression, whereas obesity downregulates its expression.

**Conclusions:**

An 8-wk HIIT can reduce hippocampal neuronal damage and CI in obese rats, and the specific mechanism may improve neuronal pathological damage and restore cognitive function by activating the SIRT1/PGC1α pathway.

## Introduction

As the leading noncommunicable disease, obesity is also a major independent risk factor for diseases such as diabetes, stroke, coronary artery disease, and cancer. The 2024 World Obesity Report states that obesity causes >120 million years of life lost annually due to noncommunicable diseases. Currently, ∼2.2 billion adults worldwide are affected by obesity, and this number is projected to reach nearly 3.3 billion by 2035, leading to a global economic loss of over $4 trillion—close to 3% of the global gross domestic product [1]. The enormous health problems and economic pressures caused by obesity have driven the deepening of research on obesity. Recent studies have found that in addition to causing traditional chronic diseases, obesity can also trigger changes in cognitive function, and there is a relationship between obesity and cognitive function [2,3]. Obesity can induce cognitive impairment (CI), and the specific mechanisms may involve obesity-induced disorders in brain oxidative phosphorylation, oxidative stress, mitochondrial dysfunction, apoptosis, and energy metabolism disorders [2,3].

As an important regulator of metabolism and cognitive function proposed in recent years, silent information regulator (SIRT)1, a histone deacetylase, plays a key role in brain health by regulating its downstream target peroxisome proliferator-activated receptor gamma coactivator (PGC)1α [[4], [5], [6], [7], [8]]. That is, the SIRT1/PGC1α pathway serves as a key network against cognitive decline. Activating this pathway can regulate oxidative stress levels, maintain mitochondrial function, and improve obesity-related neurologic issues such as neuroinflammation, thereby reducing CI [[4], [5], [6], [7], [8], [9], [10], [11]].

Exercise has been confirmed as one of the most effective means to combat obesity. Among various exercise protocols, numerous studies have demonstrated that high-intensity interval training (HIIT) is the most effective nonpharmacological approach for improving obesity-related metabolic diseases [[12], [13], [14]]. Moreover, HIIT exercise can regulate SIRT1 levels in various organs [15,16]. However, current research has rarely explored the effects of HIIT exercise on hippocampal neuronal damage and cognitive function induced by obesity, particularly the improvement of hippocampal neuronal damage and CI in obese rats by regulating the SIRT1/PGC1α pathway [[17], [18], [19]].

Therefore, in this study, obese model rats were established through high-fat diet feeding, followed by an 8-week (8-wk) HIIT intervention, to investigate the effects and mechanisms of HIIT in improving cognitive dysfunction in obese rats. This study provides a new research direction for the prevention and treatment of obesity-induced CI through exercise and suggests the need to widely expand research in obesity-related metabolic diseases.

## Methods

### Establishment and grouping of animal models

This study used 80 male SPF-grade Sprague–Dawley rats aged 5 wk as research subjects. All animals were housed in the Animal Laboratory of Beijing Sport University under standardized conditions: well-ventilated environment, temperature maintained at 20–24 °C, relative humidity of 40%–60%, and a 12-h light–dark cycle simulating natural environmental rhythms. The rats were group-housed in cages (5 rats per cage) and underwent a 1-wk adaptive feeding period with standard maintenance diet provided by Beijing Huafukang Biotechnology [License No.: SCXK (Jing) 2019-0008]. Standard rodent feed pellets and autoclaved drinking water were available ad libitum throughout the study period.

Subsequently, 40 rats were randomly selected and transitioned to a high-fat diet [D12451, supplied by Keao Xieli (Tianjin) Feed; License No.: Jin Feed Certificate (2020) 01105], whereas the remaining 40 rats continued on the standard maintenance diet. Throughout the obesity modeling phase, body weight was recorded weekly. After 8 wk, all rats underwent body weight measurement and body length assessment to compute Lee index as follows: Lee index = [body weight (g)]^1/3^ × 1000/body length (cm). When the body weight of rats fed with high-fat diet exceeded 20% of the average body weight of rats fed with regular diet and when there was a statistical difference in body weight and Lee index between the groups of rats (*P* < 0.05), they were determined to be obese rats. A total of 20 successfully modeled obese rats were ultimately selected. The modeling results are tabulated in Table 1.

**TABLE 1:** Modeling results of rats.

Group	No. of rats	Weight (g)	Body length (cm)	Lee index
Normal control rats (CG)	20	434.26 ± 43.85	23.77 ± 0.55	6087.30 ± 553.33
High-fat group (HG)	20	624.26 ± 38.61^1^	26.50 ± 0.96^1^	7862.60 ± 566.91^1^

1 *P* < 0.01 compared with CG rats.

Following the abovementioned selection criteria, high-fat group (HG) and age-matched normal control group (CG) were randomly assigned into exercise and sedentary subgroups, with 10 animals per subgroup. No significant differences were observed in baseline data between the high-fat sedentary group (HSG) and high-fat exercise group (HEG), nor between the normal sedentary group (CSG) and normal exercise group (CEG) (Table 2). Subsequently, obese rats remained on the high-fat diet, whereas normal rats continued with the standard diet, and all groups underwent an 8-wk exercise intervention protocol. This study was approved under the Certificate of Corporate Member of Beijing Laboratory Animal Industry Association (JDXT0029) and Laboratory Animal Ethics Review No. 2024068A.

**TABLE 2:** Grouping of experimental rats.

Group	No. of rats	Weight (g)	Body length (cm)	Lee index
Normal sedentary group (CSG)	10	438.53 ± 44.27	23.80 ± 0.54	6138.96 ± 566.68
Normal exercise group (CEG)	10	429.98 ± 45.37	23.73 ± 0.60	6035.64 ± 565.06
High-fat sedentary group (HSG)	10	630.95 ± 42.08	27.00 ± 1.03	7798.60 ± 572.43
High-fat exercise group (HEG)	10	617.57 ± 35.74	26.00 ± 0.58	7926.60 ± 584.55

### Randomization and blinding of animals

To ensure baseline homogeneity and reduce subjective bias, strict randomization and blinding procedures were implemented in this study.

#### Randomization procedures

Two rounds of stratified randomization were conducted for the 2 grouping stages, with all randomization performed by an independent researcher using SPSS 26.0 software and allocation concealed via encrypted animal IDs. For the first-stage diet grouping before modeling, 80 rats were stratified into 4 weight strata after 1 week of adaptive feeding, with 40 rats randomly assigned to the HG and 40 to the CG at a 1:1 ratio within each stratum; no significant baseline differences in body weight, body length, or Lee index were observed between groups (all *P* > 0.05). For the second-stage intervention grouping after successful modeling, 20 successfully modeled HG rats and 20 age-matched CG rats were stratified by body weight, then randomly allocated to exercise (E) or sedentary (S) subgroups, respectively, at a 1:1 ratio within each stratum, forming 4 subgroups (HSG, HEG, CSG, and CEG; *n* = 10 each), with no significant baseline differences in key parameters between corresponding subgroups (all *P* > 0.05).

#### Blinding procedures

Allocation concealment was ensured as randomization lists were encrypted by an independent statistician, with only encrypted IDs used in experimental records until data analysis completion. Exercise implementers were unblinded due to visible intervention differences but followed standardized protocols consistently. Researchers responsible for outcome measurement and sample analysis remained blinded to group assignments via encrypted IDs, whereas deidentified data were analyzed by a blinded statistician; group labels were matched to results after analysis only after confirmation of data integrity.

### Determination of exercise intensity

The exercise intervention used HIIT, with exercise intensity calibrated to the running speed corresponding to maximal oxygen uptake (VO_2max_). To this end, VO_2max_ testing was performed on HIIT-group rats via a modified incremental treadmill protocol for rodents [20]. Gas exchange parameters during VO_2max_ testing were measured using a gas metabolism analyzer (Columbus Instruments), with the treadmill system modularly integrated with the gas analysis module. The detailed testing protocol is outlined in Table 3.

**TABLE 3:** Test plan for VO_2max_ uptake in rats.

Grade	**Velocity (km·h⁻¹)**	**Duration (min)**
1	0.6	4
2	0.9	4
3	1.2	4
4	1.5	4
5	1.8	4
6	2.1	4
7	2.4	4
8	2.7	4
9	3.0	4

The criteria for terminating the test are that the rats cannot continue to exercise or have a plateau period of maximum oxygen uptake after receiving electric shock stimulation. Test the HIIT intervention group rats every 4 wk, record the running speed corresponding to the maximum oxygen uptake after each test, and adjust the running speed on the basis of the test results.

Abbreviation: HIIT, high-intensity interval training.

### Sports training arrangements

#### Sedentary group

No exercise training was administered.

#### HIIT exercise group

The running velocity for the HIIT intervention group was established based on VO_2max_ test results, preliminary experimental data, and existing literature. Animals in the HIIT intervention group underwent an 8-wk HIIT protocol consisting of 3 sessions per week, 36 minutes per session. VO_2max_ testing was performed every 4 wk to dynamically adjust the running speed for HIIT-trained rats. The detailed exercise protocol for the HIIT intervention group is presented in Table 4.

**TABLE 4:** High-intensity interval training program.

Group	Intensity (%VO_2max_)	Time (min)	Frequency
Warm up	50%	1	1 cycle
High-intensity exercise period	90%	2	7 cycles
Intermittent period of high-intensity exercise	65%	3	7 cycles

### Behavioral experimental testing—Morris water maze

Following the 8-wk HIIT training, the Morris water maze (MWM) paradigm was used to evaluate spatial navigation and exploratory behavior in each group, providing insights into learning memory and spatial memory capabilities. The testing protocol included 3 phases: cued learning phase, place navigation test, and spatial probe test. The cued learning phase, consisting of 4 trials, was conducted 1 d before the place navigation test to assess the rats’ ability to swim toward a visual cue (target) and acclimate to the experimental environment. During this phase, the submerged platform was marked by a distinct “flag” positioned above the water surface. For the place navigation test, over the first 5 d, rats were individually introduced into the pool facing the wall from a fixed quadrant each day, and escape latency (time to locate the platform) was recorded. If a rat failed to locate the platform within 120 s, it was manually guided to the platform, with latency recorded as the maximum 120 s. A 15-min intertrial interval was strictly maintained, and the daily escape latency was calculated as the mean of 4 consecutive trials.

Regarding spatial probe test, on day 6, the platform was removed, and rats were reintroduced into the pool from the same quadrant used in the place navigation test, facing the wall. The number of platform-crossing events within 60 s was recorded as an index of spatial memory retention.

### Animal sampling

After 24 hours of the completion of behavioral assessments, all rats were anesthetized using intraperitoneal administration of pentobarbital sodium at a dose of 50 mg/kg body weight. Brain tissue collection and perfusion fixation were then performed, with the entire sampling process carried out on ice to maintain tissue integrity.

### Tissue perfusion and paraffin sectioning

Following isoflurane anesthesia, rats underwent transcardial perfusion through the cardiac apex with 500 mL of 0.9% saline for 5 min, followed by 4% paraformaldehyde for 5 min to achieve prefixation. Brains were harvested and subjected to postfixation in 4% paraformaldehyde for 24 h and then dehydrated using a graded ethanol series (75%, 85%, 95%, and absolute ethanol, with incubation times ranging from 30 min to 2 h per concentration). Tissues were subsequently cleared in xylene and infiltrated with molten paraffin through 3 successive 1-h incubations. Embedded blocks were cooled at −20 °C, trimmed to expose the tissue surface, and sectioned into 4-μm-thick slices using a microtome. Sections were mounted on glass slides, flattened, and baked at 60 °C for long-term storage.

### Hematoxylin and eosin staining

Paraffin sections of brain tissue were subjected to deparaffinization using environmentally friendly dewaxing solutions I and II (20 min each), followed by gradient rehydration with absolute ethanol (two 5-min washes), 75% ethanol (5 min), and subsequent water rinsing. Hematoxylin and eosin staining was performed for 2–5 min, followed by differentiation with 0.1% glacial acetic acid and termination of the reaction by water washing. Sections were air dried, cleared in xylene for 10 min, and coverslipped with neutral balsam. Digital images were captured using a pathological slide scanner, and quantitative analysis was conducted using ImageJ software.

### Nissl staining

Paraffin-embedded brain sections were deparaffinized with environmentally friendly dewaxing solutions I and II (20 min each), followed by gradient rehydration through absolute ethanol (two 5-min washes), 75% ethanol (5 min), and water rinsing. Sections were stained with Nissl stain for 2–5 min, differentiated with 0.1% glacial acetic acid, and the reaction was terminated by water washing. After air drying, sections were cleared in xylene for 10 min and coverslipped with neutral balsam. Digital images were acquired using a pathological slide scanner, and quantitative analysis was performed using ImageJ software.

### Tissue RT-qPCR assay

Hippocampal tissues from each group were collected and placed into glass homogenizers. After adding 1 mL of Trizol lysis buffer, tissues were thoroughly homogenized to disrupt cells. Total RNA was extracted using the chloroform–isopropanol method, followed by reverse transcription to synthesize cDNA using reverse transcriptase. All procedures were performed on ice to maintain RNA integrity. Primers were designed based on target gene sequences retrieved from the NCBI (National Center for Biotechnology Information) database (Table 5), and RT-qPCR was conducted. Relative expression levels of target genes were calculated using the formula 2^(−ΔΔCt)^ based on the C_t_ values of each reaction well.

**TABLE 5:** Primer sequences for tissue RT-qPCR.

Gene	Forward primer	Reverse primer
*Sirt1*	AGTAACAGTGACAGTGGCACATGC	CCTCCGTCAGCTCCAGATCCTC
*Gapdh*	ACGGCAAGTTCAACGGCACAG	CGACATACTCAGCACCAGCATCAC

### Western blot analysis

Tissues of the prefrontal cortex and hippocampus from each group of rats were homogenized in liquid nitrogen, followed by the rapid addition of 1 mL of RIPA (Radioimmunoprecipitation Assay) lysis buffer supplemented with protease inhibitors to ensure complete tissue disruption. The lysates were centrifuged at 12,000*g* for 15 min at 4 °C. Four hundred microliters of the supernatant was mixed with 100 μL of 5× protein loading buffer, and the resultant mixture was subjected to protein denaturation in a 100 °C water bath for 10 min. Subsequent to SDS-PAGE electrophoresis, proteins were transferred onto PVDF (Polyvinylidene Fluoride) membranes. The membranes were blocked with 5% skim milk at room temperature for 1 h and then incubated with appropriately diluted primary antibodies (SIRT1 and PGC1α antibodies obtained from Abcam; β-actin antibody from Beyotime) at 4 °C overnight. On the following day, membranes were washed 3 times with TBST (5 min per wash) to remove unbound primary antibodies, followed by incubation with horseradish peroxidase–conjugated secondary antibodies (goat antirabbit IgG and goat antimouse IgG from Beyotime) at room temperature for 1 h. After 4 additional Tris-buffered saline Tween washes (5 min per wash), protein signals were visualized using ECL (Enhanced Chemiluminescence) chemiluminescent reagents and analyzed via a gel imaging system.

### Data processing and statistical analysis

Statistical analyses were conducted using SPSS 26.0 software, with all data representing the results of 3 independent experiments. Graphical representations were generated using Prism GraphPad 8.0 software, and a significance level of *P* < 0.05 was defined for statistical differences. The normality of data distribution was assessed via the Shapiro–Wilk test, and the homogeneity of variances was verified using Levene test. Data that conformed to normal distribution and homogeneity of variances were expressed as mean ± SD. Statistical analysis was conducted using 2-way analysis of variance (ANOVA), with obesity status and exercise intervention as between-group main effects, and their interaction effect was analyzed. In the presence of a significant interaction effect, the least significant difference test was used for simple effect analysis; in the absence of an interaction effect, main effect tests were performed. In cases where normality was violated, data were transformed using appropriate methods (e.g., logarithmic transformation) or analyzed using the nonparametric Kruskal–Wallis test as an alternative. If homogeneity of variances was violated, Welch ANOVA was applied, followed by the Games–Howell post hoc test for multiple comparisons.

## Results

### Body weight and Lee index of rats across experimental groups

Data presented in Table 6 illustrate that, following an 8-wk HIIT intervention, both body weight and Lee index of the exercised rats showed a decreasing tendency. Although the CEG demonstrated a reduction in body weight and Lee index relative to the CSG, these differences did not achieve statistical significance (*P* > 0.05). Compared with the HSG, the body weight of the HEG decreased significantly (*P* < 0.001; Cohen d = 2.64; 95% CI: 73.98, 143.95). Compared with the HSG, Lee index of the HEG decreased significantly (*P* < 0.001; Cohen d = 1.92; 95% CI: 565.49, 1545.68). This indicates that HIIT effectively reduced high-fat diet-related weight gain and obesity.

**TABLE 6:** Body weight and Lee index of rats in each experimental group following 8 weeks of high-intensity interval training intervention (*n* = 10).

Group	No. of rats	Weight (g)	Body length (cm)	Lee index
Normal sedentary group (CSG)	10	462.24 ± 25.97	24.10 ± 0.46	6395.64 ± 385.43
Normal exercise group (CEG)	10	432.88 ± 43.07	24.33 ± 0.72	5939.06 ± 645.36
High-fat sedentary group (HSG)	10	678.8 ± 56.66^1^^,^^2^	27.80 ± 1.03^1^^,^^2^	8148.08 ± 718.19^1^^,^^2^
High-fat exercise group (HEG)	10	569.84 ± 14.50^1^^,^^2^^,^^3^	26.80 ± 0.58^1^^,^^2^^,^^4^	7092.50 ± 295.10^2^^,^^3^^,^^5^

1 *P* < 0.01 compared with CSG rats.

2 *P* < 0.01 compared with CEG rats.

3 *P* < 0.01 compared with HSG rats

4 *P* < 0.05 compared with HSG rats.

5 *P* < 0.05 compared with CSG rats.

### Effects of HIIT exercise on cognitive function in obese rats

Using the MWM assay, this study compared and analyzed differences in escape latency and platform-crossing frequency among rat groups, macroscopically revealing that HIIT exercise improves cognitive performance in obese rats through behavioral changes.

#### MWM latency test results

**FIGURE 1:**
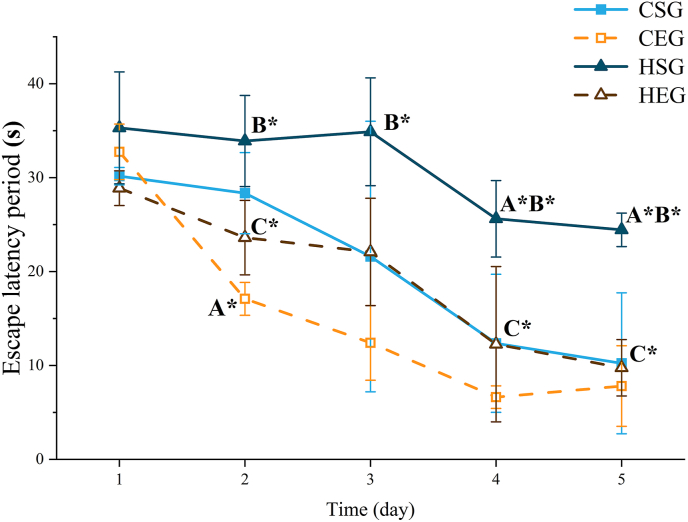
Trend of escape latency period changes in the Morris water maze test across rat groups. Letters indicate comparison groups: A, compared with CSG; B, compared with CEG; C, compared with HSG. Statistical significance: ∗*P* < 0.05; ∗∗*P* < 0.01. Combined letters (e.g., A∗B∗) indicate significance against both groups (A and B). CEG, normal exercise group; CSG, normal sedentary group; HEG, high-fat exercise group; HSG, high-fat sedentary group.

As shown in Figure 1 and Table 7, after 5 days of learning, the escape latency of all rat groups gradually decreased day by day. Compared with the CSG, the CEG exhibited a significant reduction in latency starting from day 2, whereas the HSG showed a significant increase starting from day 4. When compared with the CEG, the HSG displayed a significant increase in latency on day 2. Notably, the HEG showed a significant decrease in latency compared with the HSG on day 2.

**TABLE 7:** Alterations in escape latency of rats across experimental groups in the Morris water maze test.

Time (d)	CSG	CEG	HSG	HEG
1	30.16 ± 0.93	32.75 ± 2.96	35.30 ± 5.96	28.88 ± 1.85
2	28.35 ± 4.32	17.10 ± 1.75^A1^	33.90 ± 4.85^B1^	23.61 ± 3.96^C1^
3	21.60 ± 14.41	12.41 ± 3.98	34.88 ± 5.74^B1^	22.09 ± 5.72
4	12.36 ± 7.35	6.63 ± 1.20	25.62 ± 4.07^A1B1^	12.26 ± 8.27^C1^
5	10.23 ± 7.51	7.81 ± 4.29	24.44 ± 1.78^A1B1^	9.76 ± 3.00^C1^

Letters indicate comparison groups: A, compared with CSG; B, compared with CEG; C, compared with HSG. Combined letters (e.g., A^1^B^1^) indicate significance against both groups (A and B).

Abbreviations: CEG, normal exercise group; CSG, normal sedentary group; HEG, high-fat exercise group; HSG, high-fat sedentary group.

^1^*P* < 0.05, statistical significance.

#### MWM platform-crossing frequency results

**FIGURE 2:**
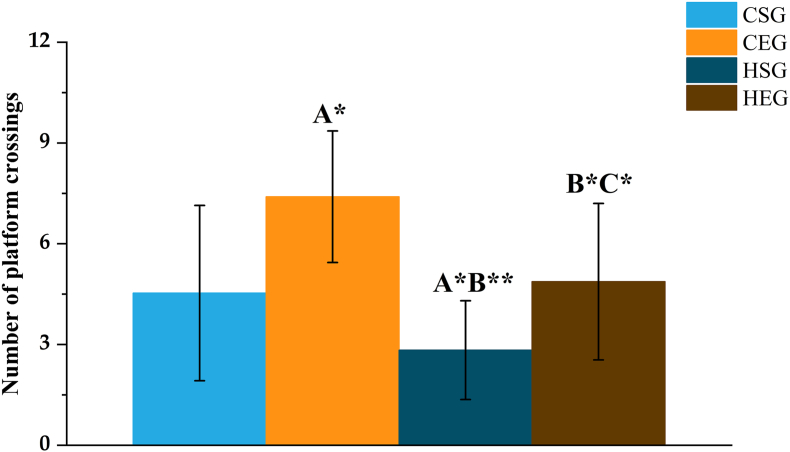
Number of platform crossings by rats in each group in the Morris water maze test. Letters indicate comparison groups: A, compared with CSG; B, compared with CEG; C, compared with HSG. Statistical significance: ∗*P* < 0.05; ∗∗*P* < 0.01. Combined letters (e.g., A∗B∗) indicate significance against both groups (A and B). CEG, normal exercise group; CSG, normal sedentary group; HEG, high-fat exercise group; HSG, high-fat sedentary group.

During the spatial probe test conducted on day 6 (Figure 2, Table 8), the CEG exhibited a significantly higher number of platform crossings than the CSG, whereas the HSG showed a significant reduction in crossing frequency. When compared with the CEG, both the HSG and HEG demonstrated significantly fewer platform crossings. Notably, the HEG displayed a significantly greater number of platform crossings than the HSG.

**TABLE 8:** Number of platform crossings by rats in each group in the Morris water maze test.

Time (d)	CSG	CEG	HSG	HEG
No. of platform crossings	4.53 ± 2.61	7.40 ± 1.96^A1^	2.83 ± 1.47^A1,B2^	4.87 ± 2.33^B1,C1^

Letters indicate comparison groups: A, compared with CSG; B, compared with CEG; C, compared with HSG. Combined letters (e.g., A^1^B^1^) indicate significance against both groups (A and B).

Abbreviations: CEG, normal exercise group; CSG, normal sedentary group; HEG, high-fat exercise group; HSG, high-fat sedentary group.

^1^*P* < 0.05, statistical significance.

^2^*P* < 0.01, statistical significance.

### The effect of HIIT on the morphology of hippocampal neurons in obese rats

**FIGURE 3:**
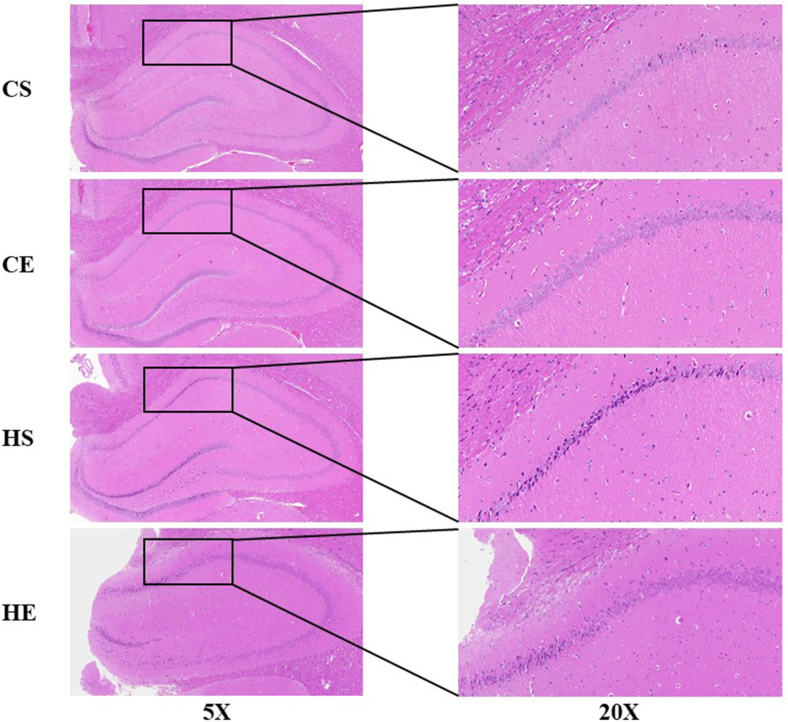
Hematoxylin and eosin staining of hippocampal tissue (×5) and hippocampal CA1 region (×20) in each group of rats. CE, normal exercise; CS, normal sedentary; HE, high-fat exercise; HS, high-fat sedentary.

**FIGURE 4:**
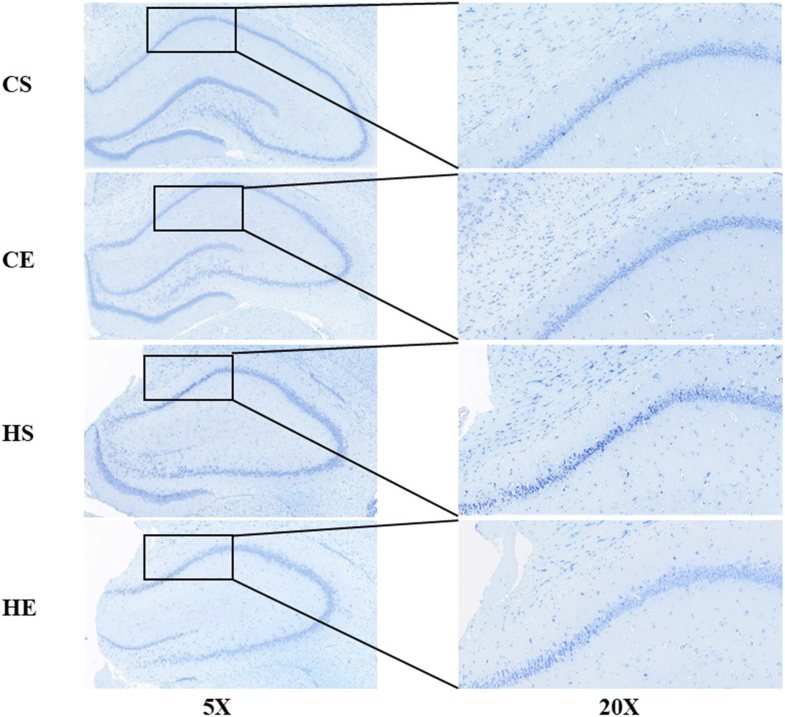
Nissl staining of hippocampal tissue (×5) and hippocampal CA1 region (×20) in each group of rats. CE, normal exercise; CS, normal sedentary; HE, high-fat exercise; HS, high-fat sedentary.

Neurodecellular atrophy and nuclear pyknosis represent the profound pathological underpinnings of cognitive dysfunction associated with obesity. This study used hematoxylin and eosin staining (Figure 3) and Nissl staining (Figure 4) to investigate neuronal alterations. Compared with the CSG, the HS group exhibited a significant reduction in the number of neurons within the CA1 region of the hippocampus, accompanied by pronounced pathological manifestations, including intensified staining, disorganized cellular arrangement, nuclear dissolution, blurred cytoplasmic decomposition, cellular atrophy, nuclear pyknosis, neurofibrillary tangles, and dissolution of Nissl bodies.

In contrast, the CEG demonstrated a significant increase in the number of neurons in the CA1 region relative to the CSG. Neurons in the CEG featured distinct, well-defined cell nuclei, clear nucleoplasmic boundaries, and orderly cellular organization. Similarly, when compared with the HSG, the HEG showed a marked increase in neuronal count, with clear nuclei, distinct nucleoplasmic demarcation, and regular cellular alignment.

Collectively, these findings indicate that a high-fat diet induced significant pathological changes in neuronal morphology in rats, whereas exercise intervention effectively mitigated these pathological alterations, highlighting the potential of HIIT as a therapeutic strategy against obesity-related neurodegeneration.

### Effects of HIIT on *Sirt1* mRNA levels in the hippocampus of obese rats

**FIGURE 5:**
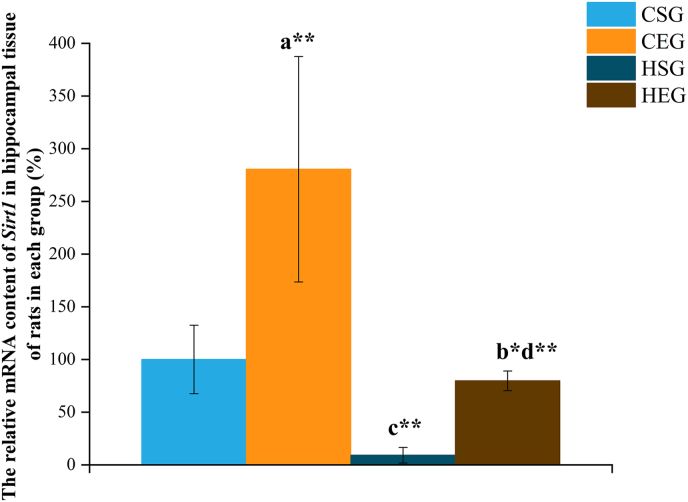
mRNA levels of Sirt1 in hippocampal tissue of rats, analyzed via a 2 × 2 factorial design (obesity status: normal-weight vs obesity; exercise status: sedentary vs exercise). The fixed obesity factor is normal weight status: a, compared with CSG. The fixed obesity factor is obese status: b, compared with HSG. The fixed exercise status is sedentary: c, compared with CSG. The fixed exercise status is exercise: d, compared with CEG. Statistical significance: ∗*P* < 0.05; ∗∗*P* < 0.01. Combined letters (e.g., a∗b∗) indicate significance against both groups (a and b). CEG, normal exercise group; CSG, normal sedentary group; HEG, high-fat exercise group; HSG, high-fat sedentary group.

The mRNA levels of the *Sirt1* in the hippocampal tissue of rats in each group were detected by RT-qPCR (Figure 5, Table 9). Two-way ANOVA for obesity and exercise intervention revealed a significant interaction effect on the hippocampal *Sirt1* mRNA levels [*F*(1,40) = 7.894; partial η^2^ = 0.208; *P* = 0.009].

**TABLE 9:** Relative mRNA content of *Sirt1* in hippocampal tissue of rats in each group (%).

Gene	CSG	CEG	HSG	HEG
*Sirt1*	100.00 ± 32.51	280.50 ± 106.96^a1^	9.13 ± 7.39^c1^	79.70 ± 9.33^b2,d1^

The fixed obesity factor is normal weight status: a, compared with CSG. The fixed obesity factor is obese status: b, compared with HSG. The fixed exercise status is sedentary: c, compared with CSG. The fixed exercise status is exercise: d, compared with CEG. Combined letters (e.g., a^2^b^2^) indicate significance against both groups (a and b).

Abbreviations: CEG, normal exercise group; CSG, normal sedentary group; HEG, high-fat exercise group; HSG, high-fat sedentary group.

^1^*P* < 0.01, statistical significance.

^2^*P* < 0.05, statistical significance.

Further simple effects analysis showed the following: under normal weight status (fixed obesity factor), a significant difference was observed in *Sirt1* mRNA levels between the CSG and CEG [*F*(1,40) = 48.660; partial η^2^ = 0.619; *P* < 0.001], with the CEG exhibiting significantly higher *Sirt1* mRNA levels than the CSG (*I-J* = 1.805; *P* < 0.001). Under obese status (fixed obesity factor), a significant difference was also detected between the HSG and HEG [*F*(1,40) = 5.781; partial η^2^ = 0.162; *P* = 0.023], with the HEG showing significantly higher *Sirt1* mRNA levels than the HSG (*I*-*J* = 0.706; *P* = 0.023).

Additionally, when exercise status was fixed, under sedentary condition, the CSG had significantly higher *Sirt1* mRNA levels than the HSG [*F*(1,40) = 13.180; partial η^2^ = 0.305; *P* = 0.001; *I*-*J* = 0.909; *P* = 0.001]. Under exercise condition, the CEG exhibited significantly higher *Sirt1* mRNA levels than the HEG [*F*(1,40) = 44.580; partial η^2^ = 0.598; *P* < 0.001; *I*-*J* = 2.008; *P* < 0.001].

### Effects of HIIT on the SIRT1/PGC1α pathway in hippocampal and prefrontal cortex tissues of obese rats

**FIGURE 6:**
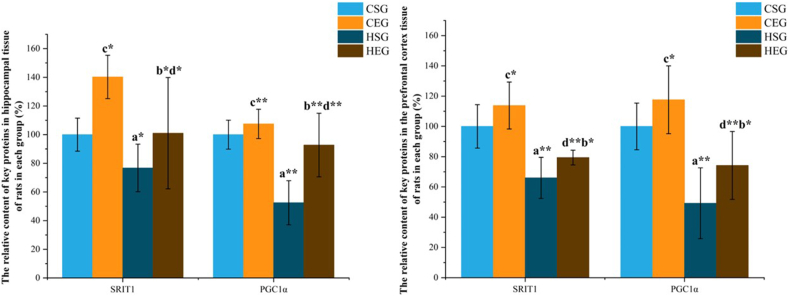
Relative expression of SIRT1/PGC1α pathway proteins in hippocampal and prefrontal cortex tissues of rats, analyzed via a 2 × 2 factorial design (obesity status: normal-weight vs obesity; exercise status: sedentary vs exercise). The main effect of obesity status is significant: a, CSG compared with HSG; b, CEG compared with HEG. The main effect of exercise status is significant: c, CSG compared with CEG; d, HSG compared with HEG. Statistical significance: ∗*P* < 0.05; ∗∗*P* < 0.01. Combined letters (e.g., a∗b∗) indicate significance against both groups (a and b). CEG, normal exercise group; CSG, normal sedentary group; HEG, high-fat exercise group; HSG, high-fat sedentary group.

**FIGURE 7:**
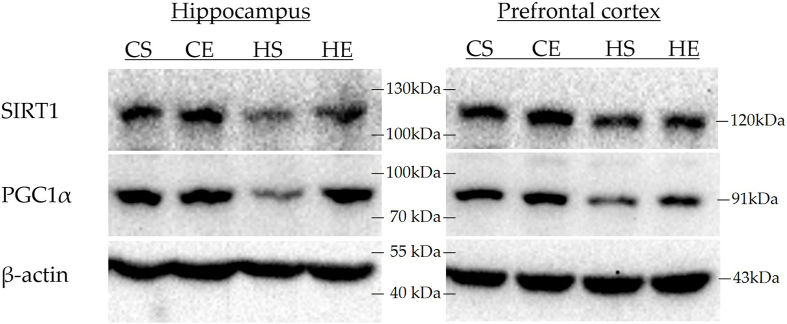
Protein grayscale expression of SIRT1/PGC1α pathway in hippocampal tissue and prefrontal cortex of rats in each group. CE, normal exercise; CS, normal sedentary; HE, high-fat exercise; HS, high-fat sedentary.

Western blot analysis was performed to determine the relative protein expression levels of SIRT1 and PGC1α in hippocampal and prefrontal cortex tissues from rats in each group (FIGURE 6, FIGURE 7, Table 10). In the hippocampus, for the SIRT1 protein, the interaction effect of obesity and exercise was not significant [*F*(1,12) = 0.477; partial η^2^ = 0.038; *P* = 0.503], whereas main effects of obesity [*F*(1,12) = 7.277; partial η^2^ = 0.377; *P* = 0.019] and exercise [*F*(1,12) = 7.750; partial η^2^ = 0.392; *P* = 0.017] were significant. Compared with the CG, the HG showed significantly downregulated SIRT1 protein levels (*P* < 0.05). Conversely, the EG exhibited significantly upregulated SIRT1 protein levels compared with the SG (*P* < 0.05). For PGC1α protein, the interaction effect of obesity and exercise was marginally nonsignificant [*F*(1,12) = 4.592; partial η^2^ = 0.277; *P* = 0.053], whereas significant main effects were observed for obesity [*F*(1,12) = 16.591; partial η^2^ = 0.580; *P* = 0.002] and exercise [*F*(1,12) = 9.762; partial η^2^ = 0.449; *P* = 0.009]. The HG had significantly lower PGC1α protein concentrations than the CG (*P* < 0.01), whereas the EG showed significantly higher PGC1α concentrations than the SG (*P* < 0.01).

**TABLE 10:** Relative protein expression content (%) of hippocampus and prefrontal cortex tissues in each group of rats.

Tissues	Protein	CSG	CEG	HSG	HEG
Hippocampus	SIRT1	100.00 ± 11.52	140.25 ± 15.12^c^^1^	76.75 ± 16.60^a^^1^	101.00 ± 38.83^b^^1^^,d^^1^
	PGC1α	100.00 ± 10.10	107.50 ± 10.21^c^^2^	52.50 ± 15.37 ^a^^2^	92.75 ± 22.17^b^^2^^,d^^2^
Prefrontal cortex	SIRT1	100.00 ± 14.353	113.80 ± 15.52^c^^1^	66.00 ± 13.58^2^	79.40 ± 4.88^b^^2^^,d^^2^
	PGC1α	100.00 ± 15.44	117.60 ± 22.42^c^^1^	49.20 ± 23.40^2^	74.20 ± 22.44^b^^2^^,d^^1^

The main effect of obesity status is significant: a, CSG compared with HSG; b, CEG compared with HEG. The main effect of exercise status is significant: c, CSG compared with CEG; d, HSG compared with HEG. Combined letters (e.g., a^1^b^1^) indicate significance against both groups (a and b).

Abbreviations: CEG, normal exercise group; CSG, normal sedentary group; HEG, high-fat exercise group; HSG, high-fat sedentary group.

1 *P* < 0.05, statistical significance.

2 *P* < 0.01, statistical significance.

In the prefrontal cortex tissue, for SIRT1 protein, no significant interaction effect of obesity and exercise was detected [*F*(1,16) = 0.001; partial η^2^ = 0.000; *P* = 0.973], but significant main effects were found for obesity [*F*(1,16) = 35.714; partial η^2^ = 0.691; *P* < 0.001] and exercise [*F*(1,16) = 5.648; partial η^2^ = 0.261; *P* = 0.030]. The HG exhibited significantly downregulated SRIT1 protein concentrations compared with the CG (*P* < 0.01), whereas the exercise group had significantly upregulated SIRT1 concentrations compared with the SG (*P* < 0.05). For PGC1α protein, the interaction effect of obesity and exercise was nonsignificant [*F*(1,16) = 0.153; partial η^2^ = 0.009; *P* = 0.701], whereas significant main effects were observed for obesity [*F*(1,16) = 24.756; partial η^2^ = 0.607; *P* < 0.001] and exercise [*F*(1,16) = 5.063; partial η^2^ = 0.240; *P* = 0.039]. Compared with the CG, the HG showed significantly lower PGC1α protein concentrations (*P* < 0.01), whereas the EG had significantly higher PGC1α concentrations than the SG (*P* < 0.05).

## Discussion

Cognitive function is an advanced psychological activity formed by the human brain after processing objective things, including perception, attention, language, calculation, visual space, learning, and memory. Cognitive function is influenced by various factors such as aging, obesity, physical activity, trauma, and infection. Obesity can lead to morphological reduction in brain volume, thinning of the cortex, white matter degeneration, loss of gyrus, and enlargement of the ventricles [21]. Pathologically, neuronal cell atrophy, dendritic degeneration, demyelination, small vessel disease, metabolic slowdown, microglial activation, and white matter lesions occur [21]. The specific pathogenesis may involve oxidative stress, decreased synaptic plasticity, chronic inflammation, and pathological brain atrophy [21].

This study findings are consistent with previous research findings, which demonstrate that obesity leads to hippocampal neuronal damage and cognitive decline in behavior. Subsequent exercise intervention experiments found that 8-wk HIIT reduced the weight and Lee index of obese rats, ameliorated their cognitive behavior and neuronal pathology levels, and increased the expression of *Sirt1* in the hippocampus of rats. Further protein-level experiments in hippocampal and prefrontal cortex tissues revealed that the SIRT1/PGC1α pathway was significantly downregulated under the influence of obesity, whereas HIIT activated the SIRT1/PGC1 α pathway to ameliorated cognitive dysfunction induced by obesity in rats.

SIRT1 is a histone deacetylase composed of 747 amino acids, including a central nuclear catalytic domain (residues 244–512), an NH2 terminal nuclear localization signal domain (residues 513–747), and a COOH (Carboxyl Group) terminal nuclear output signal domain (residues 1–180) [22,23]. SIRT1 as a histone deacetylase, has a wide range of physiological regulatory abilities and can deacetylate histones (such as H1, H3, and H4) and FOXO (Forkhead Box O) family proteins PGC1α. Nonhistone proteins such as nuclear transcription factor κB [[24], [25], [26]]. PGC1α is a downstream target of SIRT1 and can be deacetylated and activated by SIRT1. The C-terminus of PGC1α contains RNA-binding sequences and serine-enriched and arginine-enriched regions, which can interact with the chromatin remodeling complex SWI/SNF family member BAF6a, thereby activating the transcription of peroxisome and mitochondrial lipid oxidation genes [27,28]. The SIRT1/PGC1α pathway plays a crucial role in brain health, serving as a key network in combating cognitive decline. It can regulate oxidative stress levels, maintain mitochondrial function, improve age-related neurological problems such as neuropathy, and alleviate cognitive dysfunction [[4], [5], [6], [7], [8]]. In studies related to the nervous system, it has been found that the SIRT1/PGC1α pathway can promote synaptic plasticity, reduce pathological atrophy and damage in the hippocampus, enhance neuronal quantity and activity, and alleviate cognitive dysfunction regulated by oxidative stress in the brain [29]. The pathological atrophy and damage of the hippocampus caused by obesity, as well as oxidative stress caused by peroxidation, are the main causes of CI [29].

Exercise can improve CI caused by obesity, and the specific mechanism may be through regulating oxidative stress, reducing cell apoptosis, promoting myelin formation, and reducing white matter damage in the brain [30]. Research has found that exercise has a regulatory effect on the transcription and protein concentrations of SIRT1 [31,32]. HIIT exercise, as the most effective exercise program to combat obesity in recent years, can also play a particularly important regulatory role in SIRT1. It has been suggested that HIIT exercise may be more sensitive to SIRT1 regulation than other forms of exercise [15]. Kazemi et al. [15] conducted resistance exercise and HIIT exercise on obese elderly women and found that the HIIT exercise group had a greater decrease in systolic blood pressure, cholesterol, HbA1C, and fasting blood sugar and a more significant increase in SIRT1. They proposed that HIIT is one of the most effective strategies for treating metabolic syndrome in elderly women [15]. Additionally, studies have found that HIIT can improve cognitive function by promoting neural plasticity and the production of neurotrophic factors [33,34], as well as facilitating the transition of neuroglial cells from a neurotoxic phenotype to a neuroprotective phenotype [35]. Moreover, some research has highlighted that HIIT may be more effective than aerobic exercise in enhancing memory ability and cognitive function [34,36,37]. On the basis of the abovementioned evidence, this experiment reasonably speculates that HIIT may improve neuronal pathological damage and cognitive dysfunction caused by obesity through the SIRT1/PGC1α pathway, providing a new theoretical basis for improving cognitive function through exercise.

However, this study has limitations. First, only male rats were included, and sex differences may influence obesity pathogenesis, cognitive regulation, and responses to exercise. Studies have documented sex-specific disparities in metabolic regulation and neuroprotection [38,39], highlighting the need for further validation in female cohorts. Second, although the 8-wk high-fat diet rapidly induced obesity in rats, it diverges from the long-term, multifaceted high-fat dietary patterns observed in humans [40,41]. Human diets are more heterogeneous, encompassing not only high fat intake but also balanced ratios of carbohydrates, proteins, and other nutrients. Given that obesity is a chronic, progressive condition, an 8-wk intervention may not fully recapitulate the pathophysiology of human obesity, thereby limiting its clinical translatability. Finally, although the HIIT protocol in this study was established based on maximal oxygen uptake test results, prior research, and existing literature [42,43], variations in parameters (e.g., high-intensity/low-intensity ratios, duration, and interval lengths) may still influence outcomes. Furthermore, its applicability to subjects across different age groups and obesity severities, as well as the safety and efficacy of long-term adherence to this protocol, remain to be investigated in subsequent studies.

## Author contributions

The authors’ responsibilities were as follows – KC, HS: designed the research; KC, JZ, CL, RL, JM: conducted the research; KC: analyzed data, KC, HS, JZ, HW, YM: wrote the article; KC: had primary responsibility for final content; and all authors: have read and approved the final manuscript.

## Data availability

Data described in the manuscript, code book, and analytic code will be made publicly and freely available without restriction at [URL]. For additional information please see our website at https://jn.nutrition.org/content/authorinfo/.

## Funding

The work was supported by the study and research funding of Fundamental Research Funds for the Central Universities of China (2022QN011).

## Conflict of interest

The authors report no conflicts of interest.
